# Physiological-Based Cord Clamping and Early Oxygenation in Newborns With Transposition of the Great Arteries: A Quality Improvement Study Protocol

**DOI:** 10.1016/j.cjcpc.2025.10.005

**Published:** 2025-10-27

**Authors:** Jesse A. Weeda, Arjan B. te Pas, Zsuzsanna Nagy, Gábor H. Kovács, Miklós Szabó, Gergő Leipold, Nariae Baik-Schneditz, Bernhard Schwaberger, Christian Heiring, Emma Louise Malchau Carlsen, Monique C. Haak, Nico A. Blom, Stuart B. Hooper, Janneke Dekker, Roel L.F. van der Palen

**Affiliations:** a Division of Neonatology, Department of Paediatrics, Willem-Alexander Children’s Hospital, Leiden University Medical Center (LUMC), Leiden, the Netherlands; b Division of Paediatric Cardiology, Department of Paediatrics, Willem-Alexander Children’s Hospital, Leiden University Medical Center (LUMC), Leiden, the Netherlands; c Department of Obstetrics and Gynaecology, Paediatric Centre, Semmelweis University, Budapest, Hungary; d Department of Neonatology, Paediatric Centre, Semmelweis University, Budapest, Hungary; e Department of Paediatric Cardiology, Gottsegen National Cardiovascular Centre, Budapest, Hungary; f Division of Neonatology, Department of Pediatrics and Adolescent Medicine and Adolescent medicine, Medical University of Graz, Graz, Austria; g Department of Neonatal and Paediatric Intensive Care, Copenhagen University Hospital, Rigshospitalet, Copenhagen, Denmark; h Faculty of Health and Medical Sciences, University of Copenhagen, Copenhagen, Denmark; i Department of Obstetrics and Fetal Medicine, Leiden University Medical Center (LUMC), Leiden, the Netherlands; j Department of Obstetrics and Gynaecology, Monash Children’s Hospital, Melbourne, Victoria, Australia

**Keywords:** congenital heart disease, transposition of the great vessels, delivery room, umbilical cord clamping, persistent pulmonary hypertension of the newborn

## Abstract

At birth, major circulatory and pulmonary adaptations are required for a successful foetal-to-neonatal transition. In newborns with transposition of the great arteries (TGA), this transition is often impaired, leading to severe hypoxemia. This may also result in persistent pulmonary hypertension of the newborn (PPHN), worsening hypoxemia, and increasing the risk of urgent invasive interventions after birth. Physiological-based cord clamping (PBCC), delaying cord clamping until after lung aeration and ventilation are established, promotes a more stable circulatory transition and has shown benefits in both preterm and term neonates. PBCC may also provide advantages in infants with congenital heart disease. In TGA, combining PBCC with early supplemental oxygen may reduce the incidence and severity of PPHN, decrease related complications, and minimize the need for invasive interventions. To evaluate the feasibility, safety, and clinical outcomes of the stabilisation approach, we have initiated a stepwise quality improvement initiative comprising 2 sequential studies. Study phase 1, a single-centre study at Leiden University Medical Centre will assess feasibility, protocol adherence, and safety. Study phase 2, an observational cohort study across multiple European centres will evaluate clinical outcomes, focusing on incidence and severity of PPHN and the need for urgent interventions. All TGA newborns will be stabilised with an intact umbilical cord while receiving 2 L/min nasal high flow (fraction of inspired oxygen 1.0) as supplemental oxygen. Cord clamping will occur once the infant is considered stable, defined as a heart rate >100 bpm and preductal SpO_2_ >75% with supplemental oxygen. This study will inform guidelines for delivery room management and early preoperative care in TGA newborns.

## Central Illustration

**Figure:**
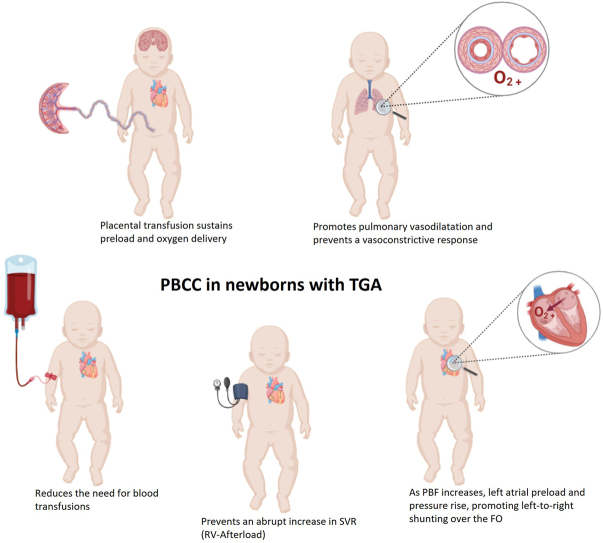

## Résumé

À la naissance, des adaptations circulatoires et pulmonaires majeures sont nécessaires pour assurer la transition du fœtus au nouveau-né. Cette transition est souvent compromise chez les nouveau-nés qui présentent une transposition des grandes artères (TGA), un état qui génère une hypoxémie sévère susceptible de provoquer une hypertension pulmonaire persistante du nouveau-né (HTPNN), une aggravation de l’hypoxémie et un risque accru d’interventions invasives urgentes après la naissance. Le clampage physiologique du cordon (CPC), qui consiste à retarder le clampage du cordon jusqu’à ce que l’aération et la ventilation pulmonaires soient établies, favorise une transition circulatoire plus stable et s’est avéré bénéfique, tant chez les prématurés et que chez les bébés nés à terme. Le CPC peut également présenter des avantages pour les nourrissons atteints d’une cardiopathie congénitale. En présence d’une TGA, le CPC combiné à une supplémentation précoce en oxygène permet de réduire l’incidence et la gravité de l’HTPNN ainsi que les complications qui lui sont associées, et de minimiser la nécessité de recourir à des interventions invasives. Nous avons entrepris un programme d’amélioration graduelle de la qualité comprenant deux études séquentielles afin d’évaluer la faisabilité, la sécurité d’utilisation et les conséquences cliniques du protocole de stabilisation. L’étude de phase I, une étude unicentrique menée au centre médical universitaire de Leiden, visera à évaluer la faisabilité, l’observance et la sécurité d’utilisation du protocole. L’étude de phase II, une étude de cohorte observationnelle menée dans plusieurs centres européens, visera à évaluer les conséquences cliniques et plus particulièrement l’incidence et la gravité de l’HTPNN ainsi que la nécessité de recourir à des interventions pratiquées d’urgence. Les nouveau-nés présentant une TGA seront tous stabilisés en maintenant leur cordon ombilical intact et recevront une supplémentation en oxygène, à raison de 2 L/min administrés à haut débit par voie nasale (fraction d’oxygène inspiré : 1,0). Le cordon sera clampé une fois que l’état du nourrisson sera jugé stable, l’état stable étant défini comme une fréquence cardiaque > 100 bpm et une saturation en oxygène (SpO_2_) préductale > 75 % avec supplémentation en oxygène. Cette étude permettra d’éclairer les lignes directrices de prise en charge en salle d’accouchement et les soins préopératoires précoces chez les nouveau-nés présentant une TGA.

At birth, major circulatory and pulmonary adaptations are required for a successful transition from foetal to neonatal circulation.^1^ Although most neonates transition seamlessly, those with congenital heart disease (CHD), such as transposition of the great arteries (TGA), often experience impaired adaptation, leading to severe hypoxemia and the need for urgent medical interventions.^2^ TGA is characterised by ventriculoarterial discordance, resulting in parallel systemic and pulmonary circulation, making postnatal arterial oxygenation dependent on foetal shunts.^3^ Effective mixing between these circulations, through an appropriately sized interatrial communication (ie, foramen ovale) or ventricular septal defect, is essential for systemic oxygenation. Increased pulmonary blood flow (PBF) and the subsequent rise in pulmonary venous return elevate left atrial pressure, promoting interatrial left-to-right shunting across the foramen ovale and enhancing oxygenation.^3^ Lung liquid clearance and aeration are the major drivers that trigger the immediate decrease in pulmonary vascular resistance (PVR) and the increase in PBF, which is then mediated by oxygen-induced pulmonary vasodilatation.^4^ The ductus arteriosus supports PBF via left-to-right shunting, increasing left atrial preload as PVR and pulmonary arterial pressure fall during neonatal adaptation.^1^^,^^4^

In neonates with TGA, particularly those with an intact ventricular septum (TGA-IVS), impaired intercirculatory mixing disrupts postnatal transition, potentially resulting in persistent pulmonary hypertension of the newborn (PPHN). PPHN further worsens hypoxemia and increases the need for invasive interventions such as mechanical ventilation and balloon atrial septostomy.^5^, ^6^, ^7^ Despite advances in prenatal detection and planned deliveries, optimising delivery room management remains challenging and underexplored in these infants. Moreover, the physiological mechanisms underlying the foetal-to-neonatal transition in TGA infants are not fully understood.^8^^,^^9^

Physiological-based cord clamping (PBCC), delaying cord clamping until after lung aeration has been established, has been shown to promote a more gradual haemodynamic transition by maintaining cardiac output, stable circulation, and oxygenation, while benefiting from optimal placental transfusion.^10^^,^^11^ Moreover, it has been shown that the vasoconstrictive response that follows immediate cord clamping (ICC) does not occur when PBCC is performed in lambs with congenital diaphragmatic hernia (CDH), and this effect also persists in the hours after birth.^12^ We therefore anticipate that PBCC, combined with early supplemental oxygen, would improve the transition in TGA neonates by reducing PVR, lowering the risk of PPHN, and minimising the need for invasive interventions, optimising early presurgical outcomes (Central Illustration). Previous studies have demonstrated that PBCC is feasible, safe, and at least as effective as standard care in preterm and term infants.^13^, ^14^, ^15^, ^16^ Moreover, a pilot randomised trial demonstrated that delayed cord clamping (DCC) in neonates with CHD, including TGA, was both feasible and safe, with a lower incidence of red blood cell transfusions compared with ICC.^17^ Given the rarity and complexity of TGA, conducting a randomised controlled trial is not feasible. Instead, we have designed a quality improvement (QI) initiative to assess the feasibility, safety, and clinical outcomes of PBCC in newborns with TGA. This approach includes PBCC with early oxygen supplementation to enhance pulmonary vasodilation and a delayed, low-dose prostaglandin regimen to limit its negative effects on respiratory drive and potential to decrease systemic vascular resistance (SVR).^8^^,^^9^ Study phase 1 comprises a single-centre feasibility and safety study at Leiden University Medical Centre (LUMC) evaluating this QI initiative. Data on protocol adherence, deviations, challenges, and safety will be used to refine the stabilisation protocol, if necessary, before its implementation across multiple European centres. Study phase 2 will evaluate the clinical outcomes of the stabilisation approach across multiple European centres.

**Central Illustration:**
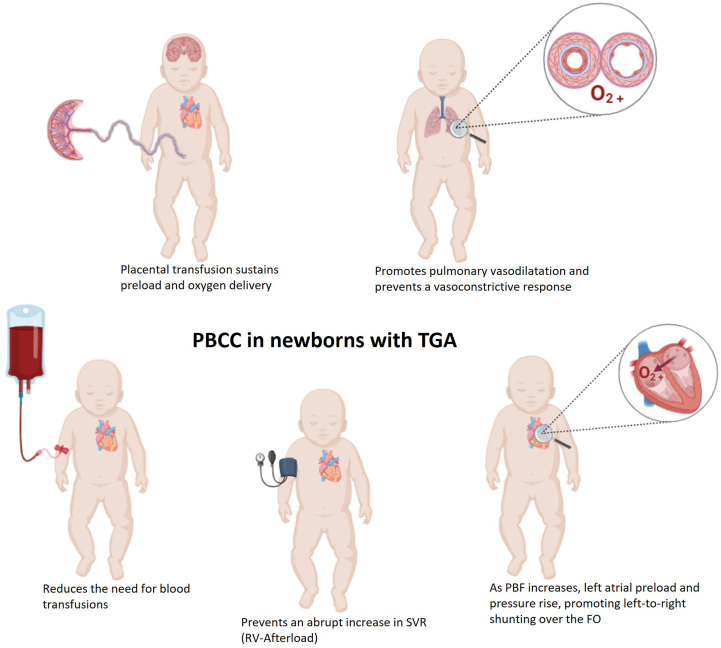
PBCC in newborns with TGA. FO, foramen ovale; PBCC, physiological-based cord clamping, PBF, pulmonary blood flow; RV, right ventricle; SVR, systemic vascular resistance. Created in https://BioRender.com.

## Methods

### Aim of the study

The objective of this study is to assess the feasibility, safety, and clinical outcomes of a novel stabilisation approach combining PBCC and early supplemental oxygen in newborns with TGA. The overall aim is to reduce the incidence and severity of PPHN and associated complications while minimising the need for early invasive interventions after birth. A visual summary of the study is provided in Video 1, view video online.

### Hypothesis

We hypothesise that a stabilisation approach combining PBCC with early supplemental oxygen is both feasible and safe, and will enhance transitional circulation in newborns with TGA. This approach is expected to reduce the incidence and severity of PPHN, minimise PPHN-related complications, decrease the need for urgent invasive interventions, and shorten intensive care unit (ICU) stay.

### Study design

**Figure 1:**
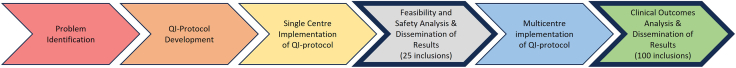
Study setup. QI, quality improvement.

This initiative is a prospective observational cohort study with a stepwise design (Fig. 1), progressing from problem identification and protocol development to implementation and evaluation.

#### Study phase 1 (single-centre feasibility and safety)

The initial phase will be carried out as a single-centre study at LUMC. Its primary aim is to assess the feasibility of the stabilisation protocol and identify potential safety concerns. This phase will allow for close monitoring of protocol adherence, deviations, and practical challenges, thereby allowing protocol refinements before expansion to multiple European centres.

#### Study phase 2 (multicentre clinical outcomes)

After completion of study phase 1, the study will extend into a multicentre observational cohort study across several European tertiary referral centres. The primary focus of this phase is the evaluation and description of clinical outcomes in an international cohort of neonates with TGA.

The study adheres to the SPIRIT 2022 guidelines for feasibility trials.^18^ The total patient enrolment period will span 4 years, with ongoing evaluation at each phase to inform the next steps.

### Study setting

Patient recruitment will take place at tertiary referral centres equipped with an obstetric high-care unit, a level 3 neonatal ICU, a paediatric cardiology department, and paediatric cardiac surgery facilities. After the initial feasibility and safety assessment, the study will expand to multiple European centres (Fig. 1), initially involving the following centres:

• LUMC (Leiden, the Netherlands)
• Semmelweis University Hospital (Budapest, Hungary)
• Copenhagen University Hospital, Rigshospitalet (Copenhagen, Denmark)
• Graz University Hospital (Graz, Austria)

### Study population

The study includes all prenatally diagnosed newborns with TGA (with or without ventricular septal defect) delivered at participating centres. Infants with complex TGA (ie, TGA with aortic coarctation, left ventricular outflow tract obstruction, or Taussig-Bing anomaly) are excluded.

### Study interventions

**Figure 2:**
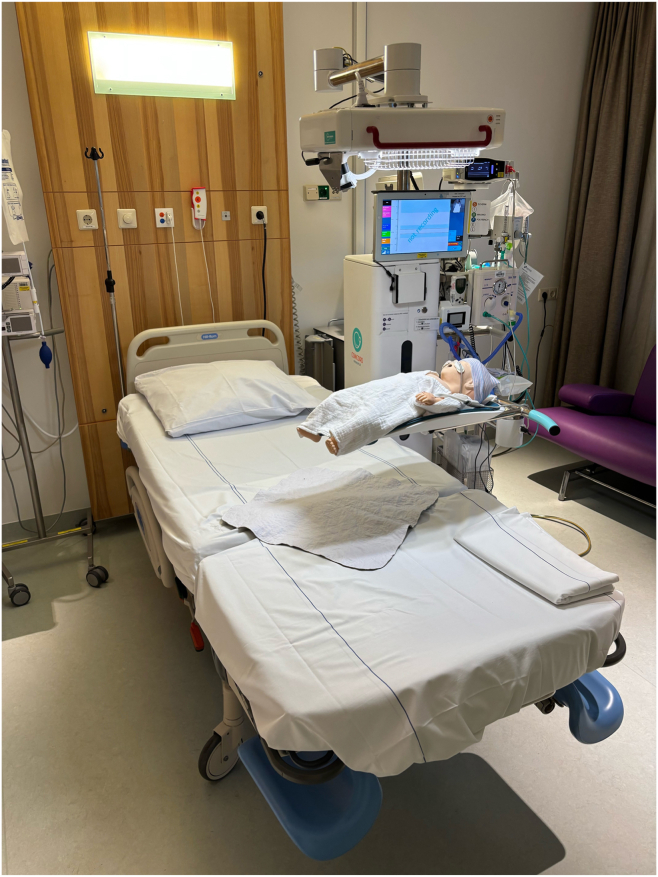
Concord resuscitation table. Example of the delivery room setup, using the Concord resuscitation table.

**Figure 3:**
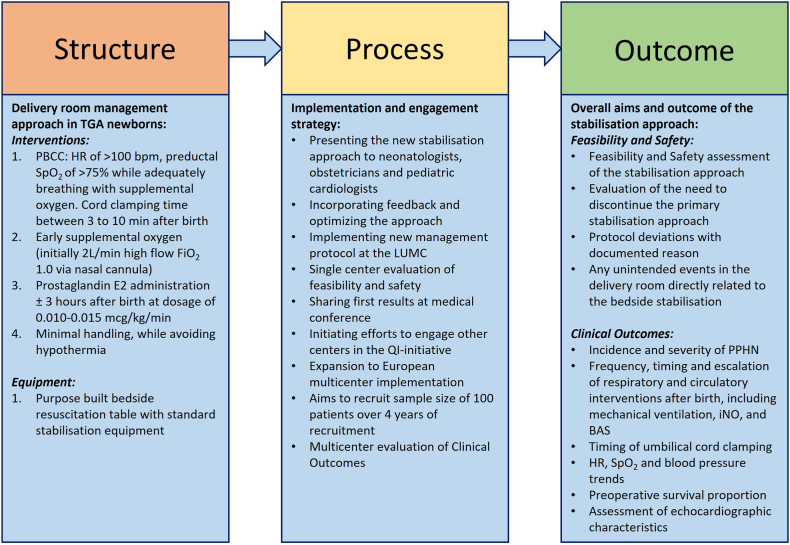
Study framework. BAS, balloon atrial septostomy; FiO_2_, fraction of inspired oxygen; HR, heart rate; iNO, inhaled nitric oxide; LUMC, Leiden University Medical Centre; PBCC, physiological-based cord clamping; PPHN, persistent pulmonary hypertension of the newborn; QI, quality improvement; SpO_2_, saturation; TGA, transposition of the great arteries.

The mode of delivery will be determined based on obstetric indications and local clinical practice. TGA newborns will undergo stabilisation on purpose-built resuscitation tables, fully equipped for bedside stabilisation and resuscitation (Fig. 2). Stabilisation will adhere to local resuscitation guidelines, with the following additions as described in the study framework (Fig. 3):

(1) *PBCC:* Based on the methods previously described by Knol et al.*,*^19^ umbilical cord clamping will be performed after stabilisation, defined as a stable clinical condition with adequate or optimised respiratory support and a heart rate >100 bpm. However, we will use a lower preductal oxygen saturation cutoff of at least 75% (SpO_2_ >75%), along with additional supplemental oxygen, due to the unique TGA physiology. The adjustments align with international clinical guidelines for managing TGA-IVS.^20^ The cord will be clamped between 3 and 10 minutes after birth.
(2) Respiratory support: Supplemental oxygen will be initiated immediately after birth, using nasal high flow at 2 L/min with a fraction of inspired oxygen of 1.0. Early administration of supplemental oxygen aims to promote further pulmonary vasodilation, thereby reducing PVR and increasing PBF.^1^^,^^4^ The supplemental oxygen will be continued until the newborn is admitted to the (neonatal) ICU. Thereafter, it will be gradually reduced by 10% every 30 minutes, provided the clinical condition remains stable, with echocardiography performed at admission and repeated if clinically indicated, until fraction of inspired oxygen reaches 0.21. The guidelines for managing TGA-IVS recommend maintaining SpO_2_ within the target range of 75%-85%.^20^ If SpO_2_ exceeds the upper target of 85%, FiO_2_ will continue to be titrated gradually at the same rate to avoid sudden hypoxemia and pulmonary vasoconstriction.^21^ If needed, respiratory support can be increased at the discretion of the treating team, in accordance with local guidelines. Given the unique parallel circulatory physiology in TGA and the relatively low-flow oxygen therapy via high-flow nasal cannula (2 L/min), systemic hyperoxia is unlikely.
(3) *Prostaglandin E2:* Prostaglandin E2 administration will be initiated approximately 3 hours after birth, following (neonatal) ICU admission and echocardiographic evaluation. The initial dosage will be approximately 0.0125 mcg/kg/min (range, 0.010-0.015 mcg/kg/min), chosen to minimise side effects, such as decreased SVR and suppression of spontaneous breathing during the transitional phase. If echocardiography identifies ductal constriction during the first examination, prostaglandin administration will be initiated sooner and/or at a higher dosage.^22^

After stabilisation of the neonate, skin-to-skin contact with the mother (for approximately 15 minutes) is encouraged to promote maternal-infant bonding, with continuous preductal saturation monitoring and additional supplemental oxygen. During ICU admission, local routine management protocols will be adhered to, with particular attention given to general preventative measures to avoid pulmonary vasoconstriction, such as preventing hypothermia. In addition, minimal handling will be implemented to further reduce stress-related pulmonary vasoconstriction and fluctuations in oxygenation.

### Study procedures

All neonatal caregivers participating in this study are trained and accredited in neonatal resuscitation at bedside, including bedside stabilisation with PBCC. Stabilisation occurs on dedicated resuscitation tables (Fig. 2) designed to support cardiorespiratory stabilisation while the cord remains intact. A standard table setup is always available as backup. If needed, the attending neonatologist or obstetrician can decide to refrain from PBCC at any time if maternal or neonatal safety is at risk, prematurely clamp the umbilical cord, and revert to local resuscitation guidelines. All indications of early clamping (<3 minutes after birth) will be documented. After stabilisation or on ICU admission, venous access is established to enable medication administration, and arterial access is established when clinically indicated or per local protocol to allow accurate monitoring of oxygenation and metabolic status.^20^

For this study, infants will be classified as having PPHN if treated with inhaled nitric oxide (iNO) for >12 hours. The decision to initiate iNO and to assess PPHN severity will be based on a combination of pre- and postductal SpO_2_ differences (>10%) and echocardiographic findings (including ductal shunt direction and flow during systole).^23^

### Primary outcome

#### Study phase 1

Feasibility and safety of the QI-management protocol will be assessed, including the need to discontinue the primary stabilisation approach, any protocol deviations with documented reason, and the number of adverse events in the delivery room. Safety assessments are based on any unintended events directly related to the bedside stabilisation and neonatal parameters, including:

• Temperature at ICU admission
• Peak haemoglobin level and the presence of polycythaemia (venous haematocrit >0.65 L/L) within 24 hours of age
• Highest bilirubin level recorded during ICU admission and the need for phototherapy and/or exchange transfusion for hyperbilirubinaemia within 7 days after birth or before neonatal arterial switch operation)
  Maternal parameters include estimated blood loss.

#### Study phase 2

The primary clinical outcomes are the incidence and severity of PPHN, assessed by pre- and postductal SpO_2_ differences, echocardiographic findings (continuous right-to-left shunting across the ductus arteriosus from the pulmonary to the systemic circulation, or a predominant right-to-left shunt throughout systole), and the frequency, timing, and escalation of invasive respiratory and circulatory interventions.

### Secondary outcome

Secondary outcome measures for both study phase 1 and study phase 2 include:

• Timing of umbilical cord clamping: Measured in minutes after birth.
• Trends in vital parameters: Heart rate, pre- and postductal SpO_2_ and blood pressure measurements will be averaged per minute and analysed for the first 72 hours after birth.
• Respiratory and circulatory interventions: Frequency and types of respiratory and circulatory interventions after birth, including noninvasive and invasive respiratory interventions, administration of iNO administration, balloon atrial septostomy, and extracorporeal membrane oxygenation.
• Survival proportion: Preoperative survival (ie, before arterial switch operation).
• **Pressure differences across the ductus arteriosus**: Ductus arteriosus pressure gradient, estimated from Doppler-derived velocities measured by echocardiography during the cardiac cycle over the first 72 hours after birth, with measurements obtained at admission and at least once every 24 hours.

### Additional neonatal outcomes for study phase 2:

• Neonatal parameters as described in phase 1, including infant temperature at ICU admission, highest infant haemoglobin level, and presence of polycythaemia; highest bilirubin level/need for phototherapy or exchange transfusion
• Apgar scores at 1, 5, and 10 minutes after birth
• Possibility, timing, and duration of skin-to-skin contact between the mother and infant

#### Baseline characteristics for both studies include:

• Gestational age
• Biological sex at birth
• Birth weight

### Maternal outcomes

Maternal outcomes will also be assessed, including estimated total blood loss, placental weight, and surgical site infection after caesarean section.

### Sample size

Patient enrolment will span 4 years, aiming to include 25 newborns in study phase 1 and 100 newborns across all centres in study phase 2. The sample size estimation is based on feasibility considerations and expected birth rates at the participating centres over the 4-year study period. As this is a prospective, descriptive, observational study design, no formal sample size calculation was performed.

### Adverse event reporting

Infants with CHD, such as TGA, are inherently at high risk for serious complications.^3^ Previous studies have shown PBCC to be feasible and safe in preterm and term infants.^13^, ^14^, ^15^, ^16^ All serious adverse events directly related to the stabilisation approach will be systematically recorded in case report forms and evaluated in both studies and presented to ensure comprehensive safety monitoring. Any unexpected or unusual serious adverse events observed during the single-centre feasibility study will be reviewed, and the protocol may be modified or the study terminated if deemed necessary before implementing the protocol in multiple European centres.

### Statistical analysis

Feasibility will be assessed by the proportion of successful stabilisation (without protocol deviations), protocol adherence, and stabilisation-related adverse events, whereas safety will be evaluated based on the occurrence and incidence of serious adverse events. Descriptive clinical outcomes in study phase 2 include the incidence and severity of PPHN; the frequency, timing, and escalation of invasive respiratory and circulatory interventions; and the occurrence of adverse events directly related to the stabilisation approach. Outcomes will be summarised using descriptive statistics and compared with existing literature. Categorical variables will be reported as frequencies and percentages, and continuous variables will be reported as means ± standard deviations or medians with interquartile ranges, depending on distribution. Within the study cohort, comparative analyses between subgroups (eg, TGA with intact ventricular septum vs TGA with ventricular septal defect and infants with vs without PPHN) will be conducted using χ^2^ or Fisher exact tests for categorical variables and independent samples *t* tests or Mann-Whitney *U* tests for continuous variables, as appropriate. Missing data will be handled appropriately and reported. A *P* value of <0.05 will be considered statistically significant. As this is an observational cohort study, no multiplicity adjustments, stopping guidelines, or formal interim analyses for early termination are planned.

### Data handling and study monitoring

To ensure data quality, clinical staff involved in data collection will be trained in protocol adherence and documentation procedures. Data handling will comply with Good Clinical Practice guidelines. All data will be systematically recorded in an Electronic Clinical Research Form and stored in a secure, password-protected database. The Castor EDC platform (www.castoredc.com) will be used for database management, and data will be pseudonymised and coded for individual specificity. Source documents will be archived for a minimum of 15 years in compliance with the General Data Protection Regulation. Preclinical data, including echocardiographic measurements, will be stored in a dedicated secure database. Data files and statistical analyses will be backed up and linked to metadata to ensure traceability and reproducibility. Access to data will be restricted to authorised personnel, and a subject identification code list will be maintained separately at LUMC’s Clinical Trial Unit, protected by password encryption. Monitoring and auditing activities will be conducted at each participating centre in accordance with national regulations and institutional requirements, as deemed necessary. Any protocol modifications during the study period will be communicated to all relevant parties in accordance with applicable local regulations.

### Ethical considerations

The study will be conducted in accordance with the principles of the Declaration of Helsinki and applicable national laws and regulations in each participating country. The protocol was reviewed and approved by the local ethical committees and/or scientific boards of the participating centres, and written informed consent was obtained from the legal guardians for participation in the study and/or the use of clinical data, where required. We do not anticipate additional risks, as the resuscitation tables are fully equipped for stabilisation and resuscitation at the bedside. PBCC has previously been performed in preterm and term infants, demonstrating both its feasibility and safety.^13^, ^14^, ^15^, ^16^ In addition, the infant may benefit from the mother’s presence during stabilisation and skin-to-skin contact. Parents will be informed about the procedures during the antenatal period.

### Patient consent

Written informed consent for study participation and/or the use of clinical data was obtained from the legal guardians of all newborns in accordance with the ethical approvals described in Ethical Considerations.

### Patient confidentiality

The principal investigator and researchers will preserve the confidentiality of participants taking part in the study in line with the Data Protection Act. Data held centrally by the principal investigator will be pseudonymised where feasible and identified by a unique code. Documents that are not pseudonymised will be kept in a strictly confidential file by the principal investigator.

### Timeline and dissemination of results

This article describes version 2.0 of the study protocol, finalised in September 2025. The study follows the SPIRIT 2022 guidelines (Supplemental Fig. S1 and Table S1). The implementation plan and engagement strategy are shown in Figures 1 and 3.

## Discussion

This study evaluates the feasibility, safety, and clinical outcomes of a novel stabilisation approach combining PBCC and early supplemental oxygen in newborns with TGA. By optimising neonatal transition, this study aims to reduce the incidence and severity of PPHN, minimise associated complications, and decrease the need for early invasive interventions after birth. The benefits of DCC in term and preterm infants without CHD are well established.^13^, ^14^, ^15^, ^16^ However, newborns with CHD are often excluded from delivery room studies, leaving limited evidence regarding the safety and potential benefits of DCC or PBCC in this population.^24^^,^^25^ Specifically in TGA, ICC is routinely performed given the anticipated need for potential urgent postnatal interventions. However, ICC abruptly increases SVR and afterload by removing the low-resistance placental circulation, while simultaneously reducing PBF and pulmonary venous return. Together, these changes result in a decrease in cardiac output.^4^^,^^26^ In TGA newborns, ICC may lead to haemodynamic instability and a prolonged period of hypoxia, potentially triggering a vasoconstrictive response and contributing to the development of PPHN.^2^^,^^25^ Studies suggest that foetuses with TGA may have abnormal pulmonary vascular development due to chronically increased oxygen content in the pulmonary artery, predisposing them to vasoconstrictive responses after birth. Histologic studies suggest features such as increased muscularisation of the pulmonary arteries, intimal proliferation, and elevated expression of mediators like endothelin-1, prostacyclin, nitric oxide, and vascular endothelial growth factor.^27^, ^28^, ^29^, ^30^ In CDH, an abnormal vascular development is also observed, where lung hypoplasia and increased vascular tone can contribute to PPHN. However, in a preclinical lamb model, it has been shown that the vasoconstrictive response that follows ICC does not occur when PBCC is performed in lambs with CDH, and this effect also persisted in the hours thereafter.^12^ Moreover, a clinical study in newborns with CDH demonstrated that intact cord resuscitation was associated with improved Apgar scores, higher mean arterial pressures, and better early blood gas values, suggesting that this approach may facilitate cardiorespiratory transition at birth in infants with CDH.^31^ We therefore anticipate that PBCC, combined with early supplemental oxygen, will improve the transition in TGA neonates, potentially reducing the incidence and severity of PPHN, minimising the need for invasive interventions, and improving early presurgical outcomes. For these infants, PBCC may provide cardiopulmonary stability during the transition from foetal to neonatal life while ensuring optimal placental transfusion. PBCC may prevent the reflex vasoconstriction in both systemic and pulmonary circulations that is typically induced by the sudden increase in SVR after ICC. Therefore, PBCC may help stabilise circulatory haemodynamics, reduce the risk of PPHN, and improve the overall postnatal transition for neonates with TGA.

In our study, PBCC is combined with early supplemental oxygen, which is expected to promote pulmonary vasodilation, thereby reducing PVR and increasing PBF. In TGA, the parallel circulatory physiology causes oxygenated blood to recirculate primarily within the pulmonary circuit. Therefore, systemic PaO_2_ should only modestly increase, even at FiO_2_ 1.0. This proposed management protocol represents a conservative, guideline-supported strategy to enhance pulmonary vasodilation and support systemic oxygen delivery immediately after birth.^20^^,^^32^^,^^33^ In addition, delaying prostaglandin infusion at a lower dose than often used may limit its adverse effects on respiratory drive and SVR in the first hours after delivery. The stabilisation criteria applied are based on those previously established for PBCC in preterm infants.^19^ However, given the unique physiological characteristics of TGA, we will adopt a lower preductal oxygen saturation cutoff (SpO_2_ >75%) in our study. These adjustments align with the international clinical guidelines for managing TGA-IVS.^20^

This approach could also transform perinatal management for other CHDs, improving bonding and stability for these newborns and facilitating broader application across centres.

### Limitations

This study focuses on newborns with TGA, which may limit the generalisability of the results to the broader CHD population. The target sample size is 25 infants for study phase 1 and 100 infants across all centres for study phase 2. This sample size was determined based on feasibility considerations rather than a formal power calculation. Although this design allows for a preliminary evaluation of the clinical outcomes of the stabilisation approach, multicentre studies with larger sample sizes will be required to draw definitive conclusions regarding its effectiveness.

## Conclusions

This prospective observational cohort study, consisting of 2 sequential study phases, evaluates the feasibility, safety, and clinical outcomes of a novel stabilisation approach combining PBCC and early supplemental oxygen in newborns with TGA. This approach may aid in stabilising the cardiopulmonary transition from foetal to neonatal life, reduce the incidence and severity of PPHN, minimise PPHN-related complications, and decrease the need for early invasive interventions. The findings of this study will inform future guidelines for delivery room management and early preoperative care in this population.

## Supplementary data

Supplementary material

Video 1 Study introduction video. This video provides an overview of the study, including rationale, objectives, and design of the proposed quality improvement initiative.
