# Supplementary material for: Physiological-Based Cord Clamping and Early Oxygenation in Newborns With Transposition of the Great Arteries: A Quality Improvement Study Protocol
Source: CJC Pediatr Congenit Heart Dis. 2025 Oct 27;5(3):136–44. doi: 10.1016/j.cjcpc.2025.10.005 (PMC13343422; doi:10.1016/j.cjcpc.2025.10.005)
Supplement: Supplementary material [file mmc1.docx]

**Supplementary information**

**Availability of data and materials**

The data collected from the trial discussed in this manuscript will be kept and stored for at least 15 years after the study concludes, in accordance with national regulations. There are no current plans to publicly share the trial data. However, all data generated or analyzed during the trial are accessible from the corresponding author upon reasonable request.

**Consent for publication**

Not applicable.

**Acknowledgements**

We thank Dr. Mikee Ishmael Inocencio for providing the voice-over of the introduction video.

**Orcid**

Roel L.F. van der Palen: <https://orcid.org/0000-0003-3640-603X>

Jesse A. Weeda: <https://orcid.org/0009-0007-4262-1808>

**Supplementary Figures**

**Supplemental Figure S1: Standard protocol items for Interventional clinical *Trials* (SPIRIT)**

|  | **STUDY PERIOD** | | | | |
| --- | --- | --- | --- | --- | --- |
|  | **Prenatal** | | **Birth, Intervention & ICU admission** | | **Close-out** |
| **Timepoint** | **-t_1_**  **Prenatal** | **0**  **Prior to birth** | **t_1_**  **Birth** | **t_2_**  **ICU admission** | **t_x_**  **Arterial switch operation** |
| **Enrolment:** |  |  |  |  |  |
| Antenatal identification | X |  |  |  |  |
| **Interventions:** |  |  |  |  |  |
| * PBCC  * Early oxygen supplementation  * Prostaglandin E2 infusion with Lower dosage at later time |  |  | X |  |  |
| **Assessments:** |  |  |  |  |  |
| Baseline variables |  | X | X |  |  |
| Primary outcome |  |  | X | X |  |
| Secondary outcome |  |  | X | X |  |
| Procedure related |  |  | X |  |  |
| Maternal |  |  | X |  |  |
| Mortality |  |  |  | X | X |

*Timeline schedule of antenatal identification, intervention and assessments at all time points. ICU: Intensive Care Unit, PBCC: Physiological Based Cord Clamping*

*Figure of identification, interventions and assessments*

**Supplementary Tables**

**Supplemental Table S1: The standard protocol items recommendations for *Trials* (SPIRIT 2022) checklist**

| **Section** | **Item No.** | **SPIRIT 2013 Item** | **SPIRIT-Outcomes 2022 item** | **Location Reported^b^** |
| --- | --- | --- | --- | --- |
| **Administrative information** | | | | |
| Title | 1 | Descriptive title identifying the study design, population, interventions, and, if applicable, trial acronym | - | Page 1 |
| Trial registration | 2a | Trial identifier and registry name. If not yet registered, name of intended registry | - | NA |
|  | 2b | All items from the World Health  Organization Trial Registration Data Set | - | NA |
| Protocol version | 3 | Date and version identifier | - | Page 1  Page 9, line 244 |
| Funding | 4 | Sources and types of financial, material, and other support | - | Supplementary files |
| Roles and responsibilities | 5a | Names, affiliations, and roles of protocol contributors | - | Page 1 and  Supplementary files |
|  | 5b | Name and contact information for the trial sponsor | - | Supplementary files |
|  | 5c | Role of study sponsor and funders, if any, in study design; collection, management, analysis, and interpretation of data; writing of the report; and the decision to submit the report for publication, including whether they will have ultimate authority over any of these activities | - | NA *investigator-initiated trial* |
|  | 5d | Composition, roles, and  responsibilities of the coordinating centre, steering committee, endpoint adjudication committee, data management team, and other individuals or groups overseeing the trial, if applicable (see Item 21a for data monitoring committee) | - | Page 9, line 214-227 and  Supplementary files |
| **Introduction** | | | | |
| Background and rationale | 6a | Description of research question and justification for undertaking the trial, including summary of relevant studies (published and unpublished) examining benefits and harms for each intervention | - | Page 3-4, line 15-43 |
|  | 6b | Explanation for choice of comparators | - | Page 9, line 200-204 |
| Objectives | 7 | Specific objectives or hypotheses | - | Page 4, line 45-49  Page 4, line 51-55 |

| **Section** | **Item No.** | **SPIRIT 2013 Item** | **SPIRIT-Outcomes 2022 item** | **Location Reported^b^** |
| --- | --- | --- | --- | --- |
| Trial design | 8 | Description of trial design including type of trial (eg, parallel group, crossover, factorial, single group), allocation ratio, and framework (eg, superiority, equivalence, noninferiority, exploratory) | - | Page 4, line 56-70 |
| **Methods: Participants, interventions, and outcomes** | | | | |
| Study setting | 9 | Description of study settings (eg, community clinic, academic hospital) and list of countries where data will be collected. Reference to where list of study sites can be obtained | - | Page 5, line 71-79 |
| Eligibility criteria | 10 | Inclusion and exclusion criteria for participants. If applicable, eligibility criteria for study centres and individuals who will perform the interventions (eg, surgeons, psychotherapists) | - | Page 5, line 80-83 |
| Interventions | 11a | Interventions for each group with  sufficient detail to allow replication, including how and when they will be administered (for specific guidance see TIDieR checklist and guide) | - | Page 5-6, line 84-123 |
|  | 11b | Criteria for discontinuing or modifying allocated interventions for a given trial participant (eg, drug dose change in response to harms, participant request, or improving/worsening disease) | - | Page 6, line 128-131  Page 9, line 189-196 |
|  | 11c | Strategies to improve adherence to intervention protocols, and any procedures for monitoring adherence (eg, drug tablet return, laboratory tests) | - | NA |
|  | 11d | Relevant concomitant care and interventions that are permitted or prohibited during the trial | - | Page 6, line 117-123 |
| Outcomes | 12 | Primary, secondary, and other outcomes, including the specific measurement variable (eg, systolic blood pressure), analysis metric (eg, change from baseline, final value, time to event), method of aggregation (eg, median, proportion), and time point for each outcome. Explanation of the clinical relevance of chosen efficacy and harm outcomes is strongly recommended | - | Page 7-8, line 157-182 |

| **Section** | **Item No.** | **SPIRIT 2013 Item** | **SPIRIT-Outcomes 2022 item** | **Location Reported^b^** |
| --- | --- | --- | --- | --- |
|  | 12.1 |  | Provide a rationale for the selection of the domain for the trials primary outcome | Page 7, line 139-155 |
|  | 12.2 |  | If the analysis metric for the primary outcome represents within-participant change, define and justify the minimal important change in individuals | NA |
|  | 12.3 |  | If the outcome data collected are continuous but will be analyzed as categorical (method of aggregation), specify the cutoff values to be used | NA |
|  | 12.4 |  | If outcome assessments will be performed at several time points after randomization, state the time points that will be used for analysis | Page 7-8, line 156-182 |
|  | 12.5 |  | If a composite outcome is used, define all individual components of the composite outcome | Page 7, line 139-155 |
| Participant timeline | 13 | Time schedule of enrolment, interventions (including any runins and washouts), assessments, and visits for participants. A schematic diagram is highly recommended (see Figure) | - | Page 5, line 69-70  Page 8, line 183-188 |
| Sample size | 14 | Estimated number of participants needed to achieve study objectives and how it was determined, including clinical and statistical assumptions supporting any sample size calculations | - | Page 8, line 183-188 |
|  | 14.1 |  | Define and justify the target difference between treatment groups (eg, the minimal important difference) | NA |
| Recruitment | 15 | Strategies for achieving adequate participant enrolment to reach target sample size | - | Page 8, line 183-188 |
| **Methods: Assignment of interventions (for controlled trials)** | | | | |
| Allocation: | | | | |
| Sequence generation | 16a | Method of generating the allocation sequence (eg, computer-generated random numbers), and list of any factors for stratification. To reduce predictability of a random sequence, details of any planned restriction (eg, blocking) should be provided in a separate document that is unavailable to those who enrol participants or assign interventions | - | NA |

| **Section** | **Item No.** | **SPIRIT 2013 Item** | **SPIRIT-Outcomes 2022 item** | **Location Reported^b^** |
| --- | --- | --- | --- | --- |
| Allocation concealment mechanism | 16b | Mechanism of implementing the allocation sequence (eg, central telephone; sequentially numbered, opaque, sealed envelopes), describing any steps to conceal the sequence until interventions are assigned | - | NA |
| Implementation | 16c | Who will generate the allocation sequence, who will enrol participants, and who will assign participants to interventions | - | NA |
| Blinding (masking) | 17a | Who will be blinded after assignment to interventions (eg, trial participants, care providers, outcome assessors, data analysts), and how | - | NA |
|  | 17b | If blinded, circumstances under which unblinding is permissible, and procedure for revealing a participant s allocated inter ention during the trial | - | NA |
| **Methods: Data collection, management, and analysis** | | | | |
| Data collection methods | 18a | Plans for assessment and collection of outcome, baseline, and other trial data, including any related processes to promote data  quality (eg, duplicate measurements, training of assessors) and a description of study instruments (eg, questionnaires, laboratory tests) along with their reliability and validity, if known. Reference to where data collection forms can be found, if not in the protocol | - | Page 9-10, line 214-227 |
|  | 18a.1 |  | Describe what is known about the responsiveness of the study instruments in a population similar to the study sample | NA |
|  | 18a.2 |  | Describe who will assess the outcome (eg, nurse, parent) | NA |
|  | 18b | Plans to promote participant retention and complete follow-up, including list of any outcome data to be collected for participants who discontinue or deviate from intervention protocols | - | NA |

| **Section** | **Item No.** | **SPIRIT 2013 Item** | **SPIRIT-Outcomes 2022 item** | **Location Reported^b^** |
| --- | --- | --- | --- | --- |
| Data management | 19 | Plans for data entry, coding, security, and storage, including any related processes to promote data quality (eg, double data entry; range checks for data values). Reference to where details of data management procedures can be found, if not in the protocol | - | Page 9-10, line 214-227 |
| Statistical methods | 20a | Statistical methods for analysing primary and secondary outcomes. Reference to where other details of the statistical analysis plan can be found, if not in the protocol | - | Page 9, line 197-212 |
|  | 20a.1 |  | Describe any planned methods to account for multiplicity in the analysis or interpretation of the primary and secondary outcomes (eg, coprimary outcomes, same outcome assessed at multiple time points, or subgroup analyses of an outcome) | Page 9, line 211-212 |
|  | 20b | Methods for any additional analyses (eg, subgroup and adjusted analyses) | - | Page 9, line 206-209 |
|  | 20c | Definition of analysis population relating to protocol nonadherence (eg, as randomised analysis), and any statistical methods to handle missing data  (eg, multiple imputation) | - | Page 9, line 210 |
| **Methods: Monitoring** | | | | |
| Data monitoring | 21a | Composition of data monitoring committee (DMC); summary of its role and reporting structure; statement of whether it is independent from the sponsor and competing interests; and reference to where further details about its charter can be found, if not in the protocol. Alternatively, an explanation of why a DMC is not needed | - | Page 5-6 line 56-70  Page 9 line 194-197 |
|  | 21b | Description of any interim analyses and stopping guidelines, including who will have access to these interim results and make  the final decision to terminate the trial | - | Page 9, line 191-197  Page 9 line 211-212 |
| Harms | 22 | Plans for collecting, assessing, reporting, and managing solicited and spontaneously reported adverse events and other unintended effects of trial interventions or trial conduct | - | Page 9, line 189-196 |

| **Section** | **Item No.** | **SPIRIT 2013 Item** | **SPIRIT-Outcomes 2022 item** | **Location Reported^b^** |
| --- | --- | --- | --- | --- |
| Auditing | 23 | Frequency and procedures for auditing trial conduct, if any, and whether the process will be independent from investigators and the sponsor | - | Page 10, line 224-227 |
| **Ethics and dissemination** | | | | |
| Research ethics approval | 24 | Plans for seeking research ethics committee/institutional review board (REC/IRB) approval | - | Page 10, line 228-237 |
| Protocol  amendments | 25 | Plans for communicating important protocol modifications (eg, changes to eligibility criteria, outcomes, analyses) to relevant parties (eg, investigators,  REC/IRBs, trial participants, trial registries, journals, regulators) | - | Page 10, line 226-227 |
| Consent or assent | 26a | Who will obtain informed consent or assent from potential trial participants or authorised surrogates, and how (see Item 32) | - | Page 10, line 230-233 |
|  | 26b | Additional consent provisions for collection and use of participant data and biological specimens in ancillary studies, if applicable | - | NA |
| Confidentiality | 27 | How personal information about potential and enrolled participants will be collected, shared, and maintained in order to protect confidentiality before, during, and after the trial | - | Page 10, line 238-242 |
| Declaration of interests | 28 | Financial and other competing interests for principal investigators for the overall trial and each study site | - | Supplementary files |
| Access to data | 29 | Statement of who will have access to the final trial dataset, and disclosure of contractual agreements that limit such access for investigators | - | Page 9-10, line 213-227 and Supplementary files |
| Ancillary and post-trial care | 30 | Provisions, if any, for ancillary and post-trial care, and for  compensation to those who suffer harm from trial participation | - | NA |
| Dissemination policy | 31a | Plans for investigators and sponsor to communicate trial results to participants, healthcare professionals, the public, and other relevant groups (eg, via publication, reporting in results databases, or other data sharing arrangements), including any publication restrictions | - | Page 10, line 243-246 |
|  | 31b | Authorship eligibility guidelines and any intended use of professional writers | - | Supplementary files |
| **Section** | **Item No.** | **SPIRIT 2013 Item** | **SPIRIT-Outcomes 2022 item** | **Location Reported^b^** |
|  | 31c | Plans, if any, for granting public access to the full protocol, participant-level dataset, and statistical code | - | Supplementary files |
| **Appendices** | | | | |
| Informed consent materials | 32 | Model consent form and other related documentation given to participants and authorised surrogates | - | NA |
| Biological specimens | 33 | Plans for collection, laboratory evaluation, and storage of biological specimens for genetic or molecular analysis in the current trial and for future use in ancillary studies, if applicable | - | NA |

^a^It is strongly recommended that this checklist be read in conjunction with the SPIRIT (Standard Protocol Items: Recommendations for Interventional Trials) Statement paper for important clarification on the items. Amendments to the protocol should be tracked and dated. The SPIRIT checklist is cop righted b the SPIRIT Gro p nder the Creati e Commons Attrib tion-NonCommercial-NoDeri s 3.0 Unported license and is reprod ced ith permission. ^b^Indicates page numbers and/or manuscript location: to be completed by authors.

Please cite as: Butcher NJ, Monsour A, Mew EJ, et al. Guidelines for reporting outcomes in trial protocols: the SPIRIT-Outcomes 2022 extension. JAMA. Published online December 13, 2022. doi:10.1001/jama.2022.21243
